# Supplementary material for: FriendlyClearMap: an optimized toolkit for mouse brain mapping and analysis
Source: Gigascience. 2023 May 23;12:giad035. doi: 10.1093/gigascience/giad035 (PMC10205001; doi:10.1093/gigascience/giad035)
Supplement: giad035_Response_to_Reviewer_Comments_Original_Submission [file giad035_response_to_reviewer_comments_original_submission.pdf]

Author's Response To Reviewer Comments

Close

Reviewer #1:
This Technical Note paper describes "FriendlyClearMap: An optimized toolkit for mouse brain mapping and analysis". Whereas the core concept of a data analysis tool to assist in spatial mapping of cleared mouse tissues is perfectly reasonable, there are multiple issues with the documentation that renders this toolkit very difficult to use. I detail below some of the issues I have encountered.

1. GitHub repository
The installation instructions are missing from the following GitHub repository:
* https://github.com/MoritzNegwer/FriendlyClearMap-scripts
The closest reference I could find to installation instructions is the following:
* "Please see the Appendices 1-3 of our publication for detailed instructions on how to use the pipelines. "
Step-by-step installation instructions should be included in the GitHub repository. In addition, the authors should add the protocols.io links to their GitHub repository.

Response to the reviewer: We agree with the reviewer that this lack of documentation and script availability was a very limiting factor in the reviewing process of our manuscript. We unfortunately haven’t received the required upload link to GigaDB prior to the review and therefore we could not provide the scripts. However, the revised versions encompass the correct download links, and we have used the chance for a revision to update the Github repository with improved code as well as with cross-platform installation scripts for Linux, Windows and MacOS.

2. Protocols.io
The installation instructions are missing from the following protocols.io links:
Run Clearmap 1 docker
* dx.doi.org/10.17504/protocols.io.eq2lynnkrvx9/v1
Run Clearmap 2 docker
* dx.doi.org/10.17504/protocols.io.yxmvmn9pbg3p/v1
Both of these protocols include the following instruction:
* "Then, download the docker container from our repository: XXX docker container goes here"
In the documentation, the authors need to unambiguously refer to the specific Docker container that a user needs to install for each software tool.

Response to the reviewer: As stated in reply to point 1, we unfortunately could not provide the relevant script after the submission and before reviewing, however, for the revision we have now uploaded the docker container to GigaDB and have added the correct links to protocols.io. We furthermore have added installation scripts for Docker Desktop on Windows, Linux and MacOS and have amended the installation instructions.

3. Test Data
I could not find the test data in the form of image stacks that would be needed to test the FriendlyClearMap protocols. Figure 1 refers to 16-bit TIFF image stacks, and I presume these to be the input data that is needed for the image analysis pipelines described in the manuscript. The authors should provide details of the test imaging dataset, including links if necessary to where the image stacks data can be downloaded, in the 'Data Availability' section of the manuscript.

Response to the reviewer: We have gladly added two folders to GigaDB with extensive test data for docker containers running our versions of Clearmap1 and Clearmap2. We also provide commands to use those verification data in the same folder, so that they can be run on any system that fufils the system requirements.

4. Platform / Operating Systems
In the 'Data Availability' section of the manuscript, the authors specify that the Operating Systems are "platform-independent". However, the protocols.io documents lists a set of requirements for Windows and LINUX, but not for MacOS. The authors should provide installation instructions and system requirements for MacOS.

Response to the reviewer: We thank the reviewer for this comment. In theory the initial docker containers could also run on MacOS, however for the revised manuscript we have indeed tested and confirmed that this is the case. We have amended the protocols.io to include installation and running the docker containers on MacOS as well.

I reject this manuscript on the grounds that, due to lack of appropriate documentation and installation instructions, the software tool is too difficult to use and therefore has extremely low reuse potential.

Response to the reviewer: We fully understand the reviewer's decision in respect to the conditions they met during the initial reviewing process. As stated before, during the submission process, we did not get the opportunity to provide the required scripts, data and instructions. We hope that with all the relevant material available in the revised version, the reviewer can critically assess the accessibility for non-programming scientist and scientific value of our analyses approach and datasets.

Reviewer #2:
This work (FriendlyClearMap) attempts to combine several tools such as ClearMap 1/2, BrainRender, etc., and integrate certain functions into a Docker image for the ease of use. The authors then demonstrated the use of FriendlyClearMap by analysing PV+, SST+ and VIP+ neurons. Some details comments are as below:
1/ P4, second paragraph, line 3, "vs." -> "versus".
2/ P9, third paragraph, line 8, conflict between "lastly" and "finally".
3/ P9, third paragraph, line 8, "our tool allows …".

Response to the reviewer: We gladly included all of the suggestions and would like to thank the reviewer for bringing these up.

4/ This work can be regarded as a reengineering effort based on several previous toolkits in order to facilitate the workflow of registration, segmentation, analysis, and visualization. Essentially, no new technology involved is involved in this work and no new application is enabled by FriendlyClearMap. Therefore, in order to emphasize the unique contribution of this work, the author could elaborate how this tool makes biologists' work easier.

Response to the reviewer: We thank the reviewer for those suggestions. Indeed, the intended audience of this work is the average neurobiologist, who might have limited experience with computational pipelines. By our own anecdotal evidence, most neurobiologists will be unfamiliar with Linux and maintaining a Python environment (or even command-line tools in general). This makes the use of existing pipelines extremely complicated, as those almost invariably will not run out of the box on a Windows machine.
We have rewritten the "Reuse potential" section to better convey our intentions. While we agree that no fundamentally new algorithms are presented here, we argue that making this pipeline accessible to a larger audience of scientists (without extensive experience in programming) solves a pressing need of the cleared-brain imaging community in particular.

5/ The results for Figure 2g are somewhat trivial. The authors might consider replace it with some more impressive analysis.

Response to the reviewer: We thank the reviewer for this assessment. For the initially submitted manuscript we intended demonstrating a representative showcase for comparing different mouse imaging datasets. We fully agree that a rise in PV+ cell numbers between P14 and P56 is scientifically not a surprising outcome and we have removed Figure 2g. Instead, we have introduced a new analysis script in a newly added Figure 4. This script adapts code from the Allen Brain Institute's CCF3 atlas work to create a cortical flat-map projection and allows a more detailed quantitative comparison of clusters of cortical cell

density. Using this method, we have discovered a previously unreported early PV+ cell maturation specific to the orbitofrontal cortex (Fig 4b, see also text description of the PV P14 dataset). A flattened-cortex projection also directly provides cortical depth profiles, both for the entire cortex and for any cortical area (Fig 4c). The addition of this readout is a scientifically relevant improvement of the previous state-of-the-art approach, Clearmap2's native cortical-depth map, which only allows depth profiles but no visualization as flattened-cortex map.

6/ The majority of the results are related to cell segmentation and counting. Quantitative plots/tables could be provided for more information. In addition, the accuracy of the results could also be discussed.

Response to the reviewer: We thank the reviewer for this request and have added per-region cell count tables to each of datasets (25 independent mouse brain hemispheres) on GigaDB, as well as overview tables per dataset to the revised version of our manuscript. We likewise provide the raw data for the cortical depth plots found in Fig. 4c.

7/ Last but not least, as there is no substantial novelty in the software, the authors actually could consider change the focus of the manuscript from a tool paper to a resource/results paper, emphasizing new biological findings which is obtained by using FriendlyClearMap.

Response to the reviewer: Indeed, we intend this paper to be used as a resource, both by providing a well-annotated data set and a comprehensive analysis pipeline.
We initially submitted it as a Technical Note to GigaScience, because per the author guidelines:
"Technical Notes should present an open-source software tool […] for the analysis or handling of large-scale data. Tools optimized to run in a cloud environment, virtual machine or container are of particular interest to the journal. The tool or method described may be new, or an update or adaptation of an existing one. For software, this includes new functionality, improvements in performance or its interface, or a release on a new platform". (https://academic.oup.com/gigascience/pages/technical_note)
We believe that by substantially improving two existing pipelines, we have made them more accessible to the larger neuroscience community, and therefore meets the requirements of a Technical Note.
However, we also agree with the reasoning that a Data Note is an appropriate category as well, as our imaging datasets are the first fully available cleared-brain imaging datasets for those specific cell types. We therefore decided to resubmit this paper as a Data Note.

Close
