# Supplementary material for: FriendlyClearMap: an optimized toolkit for mouse brain mapping and analysis
Source: Gigascience. 2023 May 23;12:giad035. doi: 10.1093/gigascience/giad035 (PMC10205001; doi:10.1093/gigascience/giad035)
Supplement: giad035_Reviewer_1_Report_Original_Submission — Chris Armit -- 7/4/2022 Reviewed [file giad035_reviewer_1_report_original_submission.pdf]

Reviewer Report

Title: FriendlyClearMap: An optimized toolkit for mouse brain mapping and analysis

Version: Original Submission **Date:** 7/4/2022

Reviewer name: Chris Armit

Reviewer Comments to Author:

This Technical Note paper describes "FriendlyClearMap: An optimized toolkit for mouse brain mapping and analysis". Whereas the core concept of a data analysis tool to assist in spatial mapping of cleared mouse tissues is perfectly reasonable, there are multiple issues with the documentation that renders this toolkit very difficult to use. I detail below some of the issues I have encountered.1. GitHub repositoryThe installation instructions are missing from the following GitHub repository:* https://github.com/MoritzNegwer/FriendlyClearMap-scriptsThe closest reference I could find to installation instructions is the following:* "Please see the Appendices 1-3 of our <X_upcoming> publication for detailed instructions on how to use the pipelines. <X_protocols.io goes here>"Step-by-step installation instructions should be included in the GitHub repository. In addition, the authors should add the protocols.io links to their GitHub repository.2. Protocols.ioThe installation instructions are missing from the following protocols.io links:Run Clearmap 1 docker* dx.doi.org/10.17504/protocols.io.eq2lynnkrvx9/v1Run Clearmap 2 docker* dx.doi.org/10.17504/protocols.io.yxmvmn9pbg3p/v1Both of these protocols include the following instruction:* "Then, download the docker container from our repository: XXX docker container goes here"In the documentation, the authors need to unambiguously refer to the specific Docker container that a user needs to install for each software tool.3. Test DataI could not find the test data in the form of image stacks that would be needed to test the FriendlyClearMap protocols. Figure 1 refers to 16-bit TIFF image stacks, and I presume these to be the input data that is needed for the image analysis pipelines described in the manuscript. The authors should provide details of the test imaging dataset, including links if necessary to where the image stacks data can be downloaded, in the 'Data Availability' section of the manuscript.4. Platform / Operating SystemsIn the 'Data Availability' section of the manuscript, the authors specify that the Operating Systems are "platform-independent". However, the protocols.io documents lists a set of requirements for Windows and LINUX, but not for MacOS. The authors should provide installation instructions and system requirements for MacOS.I reject this manuscript on the grounds that, due to lack of appropriate documentation and installation instructions, the software tool is too difficult to use and therefore has extremely low reuse potential.

Level of Interest

Please indicate how interesting you found the manuscript: Choose an item.

Quality of Written English

Please indicate the quality of language in the manuscript: Choose an item.

Declaration of Competing Interests

Please complete a declaration of competing interests, considering the following questions:

- Have you in the past five years received reimbursements, fees, funding, or salary from an organisation that may in any way gain or lose financially from the publication of this manuscript, either now or in the future?
- Do you hold any stocks or shares in an organisation that may in any way gain or lose financially from the publication of this manuscript, either now or in the future?
- Do you hold or are you currently applying for any patents relating to the content of the manuscript?
- Have you received reimbursements, fees, funding, or salary from an organization that holds or has applied for patents relating to the content of the manuscript?
- Do you have any other financial competing interests?
- Do you have any non-financial competing interests in relation to this paper?

If you can answer no to all of the above, write 'I declare that I have no competing interests' below. If your reply is yes to any, please give details below.

I work for GigaScience where I perform checks on the quality of image data and the image analysis approach. My funding is not dependent on the outcome of this review, and I declare that I have no competing interests.

I agree to the open peer review policy of the journal. I understand that my name will be included on my report to the authors and, if the manuscript is accepted for publication, my named report including any attachments I upload will be posted on the website along with the authors' responses. I agree for my report to be made available under an Open Access Creative Commons CC-BY license (http://creativecommons.org/licenses/by/4.0/). I understand that any comments which I do not wish to be included in my named report can be included as confidential comments to the editors, which will not be published.

Choose an item.

To further support our reviewers, we have joined with Publons, where you can gain additional credit to further highlight your hard work (see: https://publons.com/journal/530/gigascience). On publication of this paper, your review will be automatically added to Publons, you can then choose whether or not to claim your Publons credit. I understand this statement.

Yes Choose an item.
