# Supplementary material for: FriendlyClearMap: an optimized toolkit for mouse brain mapping and analysis
Source: Gigascience. 2023 May 23;12:giad035. doi: 10.1093/gigascience/giad035 (PMC10205001; doi:10.1093/gigascience/giad035)
Supplement: giad035_Reviewer_1_Report_Revision_1 — Chris Armit -- 3/3/2023 Reviewed [file giad035_reviewer_1_report_revision_1.pdf]

Reviewer Report

Title: FriendlyClearMap: An optimized toolkit for mouse brain mapping and analysis

Version: Revision 1 **Date:** 3/3/2023

Reviewer name: Chris Armit

Reviewer Comments to Author:

This Technical Note paper describes "FriendlyClearMap: An optimized toolkit for mouse brain mapping and analysis". The core concept of this toolkit is a suite of data analysis tools to assist in spatial mapping of cleared mouse tissues. The manuscript is well written, and the supporting data have been submitted to the GigaScience DataBase.I reviewed a previous version of this manuscript, and I thank the authors for addressing my comments. In particular, in my initial review, I pointed out that the installation instructions are missing from the following GitHub repository:* https://github.com/MoritzNegwer/FriendlyClearMap-scriptsIn the revised manuscript and supporting data, I thank the authors for adding protocols.io links to their GitHub repository. I confirm that the protocols.io links provide installation instructions for the various software tools on multiple platforms, and include the following details relating to system requirements.Run Clearmap 1 docker V.1dx.doi.org/10.17504/protocols.io.eq2lynnkrvx9/v1Platform: Windows / LINUXRun Clearmap 2 docker V.2dx.doi.org/10.17504/protocols.io.yxmvmn9pbg3p/v2Platform: Windows / LINUX / MacOSVisualize Data with Brainrenderdx.doi.org/10.17504/protocols.io.dm6gpbdwdlzp/v1Platform: LINUXIn addition, I thank the authors for updating the "System Requirements" section of the manuscript so that it now includes the following detail:"We verified that the Docker containers with the core FriendlyClearmap pipeline run on Linux (Ubuntu 20.04 and 22.04), Windows (Windows 10 and Windows Server on Amazon AWS) and MacOS (MacOS 12.6.2 Monterey)."I additionally confirm that I was able to run the FriendlyClearmap Docker container on MacOS Monterey 12.3 with Apple M1 Chip.

Level of Interest

Please indicate how interesting you found the manuscript: Choose an item.

Quality of Written English

Please indicate the quality of language in the manuscript: Choose an item.

Declaration of Competing Interests

Please complete a declaration of competing interests, considering the following questions:

- Have you in the past five years received reimbursements, fees, funding, or salary from an organisation that may in any way gain or lose financially from the publication of this manuscript, either now or in the future?

- Do you hold any stocks or shares in an organisation that may in any way gain or lose financially from the publication of this manuscript, either now or in the future?
- Do you hold or are you currently applying for any patents relating to the content of the manuscript?
- Have you received reimbursements, fees, funding, or salary from an organization that holds or has applied for patents relating to the content of the manuscript?
- Do you have any other financial competing interests?
- Do you have any non-financial competing interests in relation to this paper?

If you can answer no to all of the above, write 'I declare that I have no competing interests' below. If your reply is yes to any, please give details below.

I work for GigaScience where I perform checks on the quality of image data and the image analysis approach. My funding is not dependent on the outcome of this review, and I declare that I have no competing interests.

I agree to the open peer review policy of the journal. I understand that my name will be included on my report to the authors and, if the manuscript is accepted for publication, my named report including any attachments I upload will be posted on the website along with the authors' responses. I agree for my report to be made available under an Open Access Creative Commons CC-BY license (http://creativecommons.org/licenses/by/4.0/). I understand that any comments which I do not wish to be included in my named report can be included as confidential comments to the editors, which will not be published.

Choose an item.

To further support our reviewers, we have joined with Publons, where you can gain additional credit to further highlight your hard work (see: https://publons.com/journal/530/gigascience). On publication of this paper, your review will be automatically added to Publons, you can then choose whether or not to claim your Publons credit. I understand this statement.

Yes Choose an item.
