# Supplementary material for: FriendlyClearMap: an optimized toolkit for mouse brain mapping and analysis
Source: Gigascience. 2023 May 23;12:giad035. doi: 10.1093/gigascience/giad035 (PMC10205001; doi:10.1093/gigascience/giad035)
Supplement: giad035_Reviewer_2_Report_Original_Submission — Yimin Wang -- 7/18/2022 Reviewed [file giad035_reviewer_2_report_original_submission.pdf]

Reviewer Report

Title: FriendlyClearMap: An optimized toolkit for mouse brain mapping and analysis

Version: Original Submission **Date:** 7/18/2022

Reviewer name: Yimin Wang

Reviewer Comments to Author:

This work (FriendlyClearMap) attempts to combine several tools such as ClearMap 1/2, BrainRender, etc., and integrate certain functions into a Docker image for the ease of use. The authors then demonstrated the use of FriendlyClearMap by analysing PV+, SST+ and VIP+ neurons. Some details comments are as below:1/ P4, second paragraph, line 3, "vs." -> "versus".2/ P9, third paragraph, line 8, conflict between "lastly" and "finally".3/ P9, third paragraph, line 8, "our tool allows …".4/ This work can be regarded as a reengineering effort based on several previous toolkits in order to facilitate the workflow of registration, segmentation, analysis, and visualization. Essentially, no new technology involved is involved in this work and no new application is enabled by FriendlyClearMap. Therefore, in order to emphasize the unique contribution of this work, the author could elaborate how this tool makes biologists' work easier.5/ The results for Figure 2g are somewhat trivial. The authors might consider replace it with some more impressive analysis.6/ The majority of the results are related to cell segmentation and counting. Quantitative plots/tables could be provided for more information. In addition, the accuracy of the results could also be discussed.7/ Last but not least, as there is no substantial novelty in the software, the authors actually could consider change the focus of the manuscript from a tool paper to a resource/results paper, emphasizing new biological findings which is obtained by using FriendlyClearMap.

Level of Interest

Please indicate how interesting you found the manuscript: Choose an item.

Quality of Written English

Please indicate the quality of language in the manuscript: Choose an item.

Declaration of Competing Interests

Please complete a declaration of competing interests, considering the following questions:

- Have you in the past five years received reimbursements, fees, funding, or salary from an organisation that may in any way gain or lose financially from the publication of this manuscript, either now or in the future?
- Do you hold any stocks or shares in an organisation that may in any way gain or lose financially from the publication of this manuscript, either now or in the future?

- Do you hold or are you currently applying for any patents relating to the content of the manuscript?
- Have you received reimbursements, fees, funding, or salary from an organization that holds or has applied for patents relating to the content of the manuscript?
- Do you have any other financial competing interests?
- Do you have any non-financial competing interests in relation to this paper?

If you can answer no to all of the above, write 'I declare that I have no competing interests' below. If your reply is yes to any, please give details below.

N/A

I agree to the open peer review policy of the journal. I understand that my name will be included on my report to the authors and, if the manuscript is accepted for publication, my named report including any attachments I upload will be posted on the website along with the authors' responses. I agree for my report to be made available under an Open Access Creative Commons CC-BY license (http://creativecommons.org/licenses/by/4.0/). I understand that any comments which I do not wish to be included in my named report can be included as confidential comments to the editors, which will not be published.

Choose an item.

To further support our reviewers, we have joined with Publons, where you can gain additional credit to further highlight your hard work (see: https://publons.com/journal/530/gigascience). On publication of this paper, your review will be automatically added to Publons, you can then choose whether or not to claim your Publons credit. I understand this statement.

Yes Choose an item.
